# Supplementary material for: The interactome of intact mitochondria by cross-linking mass spectrometry provides evidence for coexisting respiratory supercomplexes
Source: Mol Cell Proteomics. 2017 Dec 8;17(2):216–32. doi: 10.1074/mcp.RA117.000470 (PMC5795388; doi:10.1074/mcp.RA117.000470)
Supplement: Supplemental Data [file supp_RA117.000470_133922_0_supp_23978_fzffwf.zip › spectra_annotation/mito_DR_spectra_annotation/293-1-11-1-5-1.pdf]

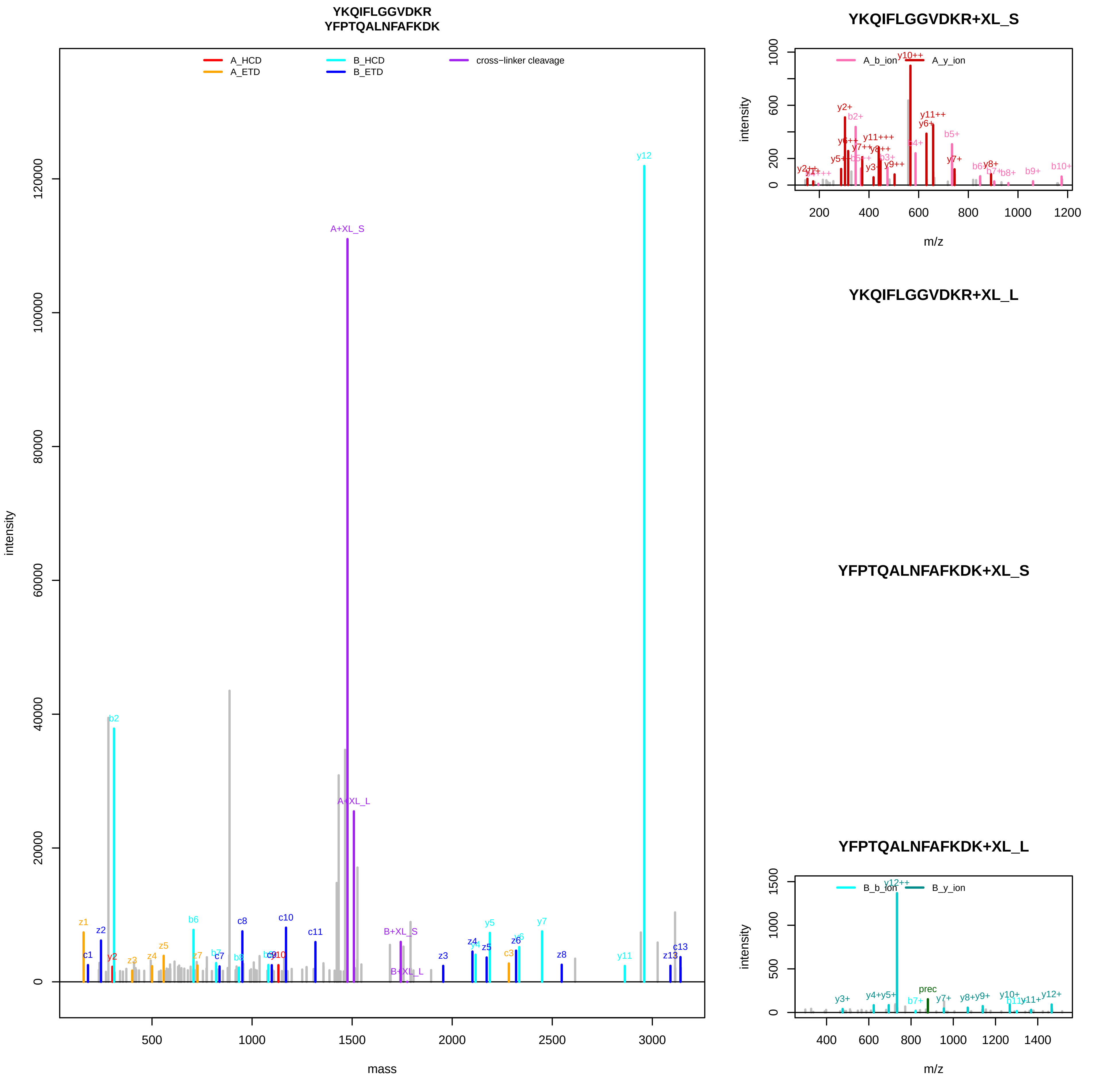

YKQIFLGGVDKR
YFPTQALNFAFKDK
A_HCD
A_ETD
B_HCD
B_ETD
cross−linker cleavage
intensity
mass
0
20000
40000
60000
80000
100000
120000
500
1000
1500
2000
2500
3000
A+XL_S
A+XL_L
B+XL_S
B+XL_L
y12
b2
b6
c8
c10
c11
y5
y7
z8
y11
z13
c13
YKQIFLGGVDKR+XL_S
A_b_ion
A_y_ion
y10++
m/z
0
200
600
1000
200
400
600
800
1000
1200
YKQIFLGGVDKR+XL_L
YFPTQALNFAFKDK+XL_S
YFPTQALNFAFKDK+XL_L
B_b_ion
B_y_ion
y12++
prec
0
500
1000
1500
400
600
800
1000
1200
1400
